# Intracoronary cytoprotective gene therapy in a dog with dilated cardiomyopathy: long term follow up

**DOI:** 10.1007/s11259-025-10691-2

**Published:** 2025-02-25

**Authors:** Paola Paradies, Lucia Carlucci, Serena Digiaro, Alessandra Recchia, Antonella Colella, Felix Woitek, Luca Lacitignola, Francesco Staffieri, Mauro Giacca, Fabio Anastasio Recchia

**Affiliations:** 1 Department of Precision and Regenerative Medicine and Ionian Area, Section of Veterinary Clinics, Campus of Veterinary Medicine, University of Bari, 70010 Valenzano, BA Italy; 2 Scuola Superiore Sant’Anna, 56100 Pisa, Italy; 3 Heart Center, Dresden at the Technical University of Dresden, 01067 Dresden, Germany; 4 School of Cardiovascular and Metabolic Medicine & Sciences and BHF Centre for Research Excellence, King’s College London, London, SE5 9NU UK; 5 Department of Medical, Surgical and Health Sciences, University of Trieste and Molecular Medicine Laboratory, International Centre for Genetic Engineering and Biotechnology (ICGEB), 34149 Trieste, Italy; 6 Institute of Clinical Physiology, National Research Council, Pisa, Italy; 7 Aging + Cardiovascular Discovery Center Lewis Katz School of Medicine, Temple University, Philadelphia, USA

**Keywords:** Dogs, Dilated cardiomyopathy, Cytoprotective, VEGF-B167

## Abstract

In large breed dogs, dilated cardiomyopathy (DCM) is the main cause of congestive heart failure (CHF) and sudden cardiac death. The underlying etiology of DCM is usually not definitively identified; however, in predisposed breeds a hereditary etiology is often suspected. Other etiologies, such as toxins and infections, have also been documented or suspected to cause DCM in dogs. Conventional drug treatment cannot reverse disease progression but can only control the signs of heart failure as they occur. Cytoprotective gene therapy with Vascular Endothelial Growth Factor-B167 (VEGF-B167) has been shown to be an effective alternative therapy that can halt disease progression in preclinical experimental studies in dogs. This study reports the long-term clinical and echocardiographic follow-up of a 6-year-old St. Bernard dog with DCM treated with intracoronary administration of VEGF-B167 gene delivered by adeno-associated viral vectors (AAV- VEGF-B167). Monitoring was performed at 1, 3, 6, 9, 12, 18, 24 and 36 months post-procedure (T0-T8) including clinical, laboratory and instrumental examinations. The dog reached T8 in good clinical condition. Comparing echocardiographic parameters from T0 to T8, ejection fraction (EF%) did not worsen, indeed showing potential improvement (30% to 38% from T0 to T8 respectively) (Simpson method). Other parameters of disease progression varied minimally over the course of the study. From T0 to T8, no relevant change in medical therapy was necessary. The dog survived 341 days from the last follow-up and died of sudden death 1436 days after the procedure (T0). A survival time of 4 years in good health is an excellent outcome suggesting a possible protective role of VEFG-B167 in slowing disease progression in this dog.

## Background

DCM is defined as a progressive left ventricular dilation and systolic dysfunction in the absence of identifiable causes (such as arrhythmia or tachycardia-induced cardiomyopathy, toxicity, myocarditis, hypothyroidism or nutritional causes). In large breed dogs DCM is the main cause of congestive heart failure (CHF) and sudden cardiac death (Walker et al. 2021; Wess 2022). Conventional pharmacological treatments aim to limit the clinical consequences of inadequate pump function, but they are unable to halt myocardial deterioration in both humans and dogs. Drugs such as pimobendan, furosemide, angiotensin-converting enzyme inhibitors, peripheral vasodilators, and other diuretics are used (Shen et al. 2022). Therefore, researchers' interest has focused on new and innovative therapies, such as gene and cell therapy, to directly treat the myocardium rather than the consequences of heart failure. The application of gene therapies to treat cardiomyopathies has recently been compared to a potential “revolution” in human medicine, changing the treatment landscape from a largely reactive treatment paradigm aimed at limiting complications to a proactive paradigm that may be curative (Argiro et al. 2024).

In this respect, gene therapy is based on a gene transfer targeting known molecular alterations that occur in cardiac cells and can’t be reversed by conventional pharmacological agents (Woitek et al. 2015). The therapeutic gene must code for a molecule that plays a critical role in the pathogenesis of the disease, promoting improved contractility and delaying progression (Greenberg 2017). Patients with DCM have been found to have cardiomyocyte changes such as fragmented DNA, implying that apoptosis is involved in the pathogenesis of DCM (Narula et al. 1996). Recently, our research group has tested gene therapy in dogs with DCM (Paradies et al. 2019). It consisted of a mini-invasive procedure of coronary artery catheterization for the infusion of adeno-associated viral vectors carrying the gene for the vascular endothelial growth factor- B167 (AAV-VEGF-B167), a known cytoprotective and anti-apoptotic factor (Li et al. 2008).

The present study reports the long-term monitoring and outcome of one of the DCM dogs treated with AAV-VEGF-B167. The patient was followed until spontaneous death and disease progression was documented by clinical, echocardiographic and laboratory examination.

## Case presentation

A 6-year-old male St. Bernard dog weighing 80 kg, was brought to the teaching hospital of the Veterinary Campus, University of Bari, for episodes of syncope, respiratory distress and abdominal enlargement. Based on clinical and instrumental exams, ventricular dilation and atrial fibrillation (AF) was diagnosed. Dietary history reported mixed commercial dry maintenance food with supplementation of homemade food. The dog was treated for AF with digoxin in monotherapy initially at 0.22 mg/m2 q12h, later adjusted based on digoxinemia, and with pimobendan 0.25 mg/kg q12h, furosemide 2 mg/kg q12h, benazepril 0.5 mg/kg q12h and spironolactone 2 mg/kg q24h reaching the complete remission of clinical signs over the following two months.

In agreement with the owner, the dog was recruited into the cytoprotective intracoronary gene therapy study (Ministerial Authorization No. 180946122 of May 2016) that included 10 dogs with clinical DCM (Paradies et al. 2019). Median survival time of the ten dogs included in the study was 500.5 days (media 546.5 days) (unpublished data). The present case report describes the patient with the longest survival time and the most complete set of measurements, including molecular ones. Diagnosis of primary DCM was based on the presence of ventricular hypokinesis in the absence of other identifiable causes or congenital or acquired cardiovascular diseases. Based on echocardiographic and ECG exams, we assessed major criteria such as left ventricular ejection fraction (EF) < 40% and fractional shortening (FS) < 20–25%, end-diastolic volume index (EDVI) > 100 ml/m^2^, end-systolic volume index (ESVI) > 30 ml/m^2^, sphericity index (SI) < 1.65, as well as other minor criteria such as the presence of an arrhythmia, atrial fibrillation, increased E-septum point (EPSS) > 7–12 mm, left atrium-to-aorta diastolic diameter ratio (LA/Ao) > 1.6. By assigning 3 points to each major criterion and 1 point to each minor criterion, a total score of 6 or more was considered pathologically significant (based on the score system proposed by Dukes-McEwan et al. 2003). Two ventricular ectopias within 5 min, measured by ECG, and/or > 100/24 h detected by Holter were considered abnormal. Dogs suspected of tachycardia-induced myocardiopathy (TCIM) were also enrolled. Exclusion criteria were concomitant diseases such as hypothyroidism, cardiopulmonary filariasis, myocarditis, pulmonary hypertension, diabetes, myasthenia gravis and pregnancy. Conventional medical therapy of DCM was used to achieve the best clinical control and was standardized for all patients at least one month prior to the gene delivery procedure.

Before the procedure, the owner signed the informed consent and blood tests (whole blood count, biochemistry, electrophoresis, urinalysis, digoxinemia and cardiac troponin measurement), X-ray, ECG and echocardiography were performed. The procedure (T0) was carried out after the reversal of clinical signs with medical therapy and when the patient was considered clinically stable.

In particular the dog of this case, at the time of the procedure was in treatment with pimobendan 0.25 mg/kg q12h, digoxin 0.17 mg/m^2^ q12h, furosemide 1.5 mg/kg q12h, benazepril 0.5 mg/kg q12h, spironolactone 2 mg/kg q24h. He was in good clinical condition with controlled AF at a mean heart rate of 73 bpm. The procedure was performed, as previously described (Woitek et al. 2015; Paradies et al. 2019), under general anesthesia with mechanical ventilation and monitoring of vital parameters (i.e. ECG, SpO2, blood pressure). The anesthesia protocol included sedation with methadone 0.3 mg/kg/IM followed by lidocaine 1 mg/kg/IV, midazolam 0.3 mg/kg/IV and propofol IV to effect. After intubation, anesthesia was maintained with isoflurane and lidocaine infusion at 30–50 μg/kg/min to prevent major arrhythmias. To obtain a complete patient’s immobility, during the catheterization of the coronary artery a bolus of rocuronium, a short-acting neuromuscular blocker (0.3 mg/kg), was administered. The Seldinger technique was used to insert an arterial line. A guiding catheter (Launcher Medtronic, USA) was introduced through the right femoral artery under fluoroscopic guidance. The left circumflex and anterior descending branches were selectively catheterized using a micro-infusion catheter (Finecross MG, Terumo Europe). 20 ml of a mixture of AAV serotype 9 (AAV-9, at a titer of 10^12^ pfu/ml) carrying VEGF-B in a buffered solution containing 3 ng/kg adenosine, 5 ng/kg substance P and 1 μg/kg nitroglycerine were slowly inoculated by a syringe pump over 20 min, followed by a 10-min flush with saline. These vasoactive substances were used to increase myocardial capillaries permeability and to reduce the flow through the coronary artery for some seconds. After injection, the catheter was removed and the right femoral artery was closed. Indeed, thanks to collateral circulation, dogs can fully meet the needs of the tissues physiologically supplied by the femoral artery.

During the intra-operative phase, the dog manifested some premature ventricular complexes (PVCs) that reversed spontaneously after catheter removal. They were probably due to the myocardial reaction to the catheter. After the procedure the dog remained in hospital under clinical and instrumental monitoring for 72 h (ECG, blood pressure measurement, blood electrolytes concentration measurement). Three to six hours after awakening from anesthesia, the dog showed a new abnormal rhythm on ECG. An idioventricular rhythm developed that reversed spontaneously within 48 h without treatment (see in Paradies et al. 2019 Fig. 2, dog 5). In addition, in the immediate postoperative period, the dog developed a haematoma at the entry site of the catheter in the right hind limb, which resolved spontaneously in a few days with a compression bandage.

Antimicrobial therapy was prescribed at hospital discharge, (ceftriaxone 20 mg/kg BID for 6–7 days) along with cardiologic pre-procedure therapy. One week after the procedure (first post procedure check) the clinical and haemato-chemical conditions of the dog were similar to those in the pre-procedure phase.

**Fig. 1:**
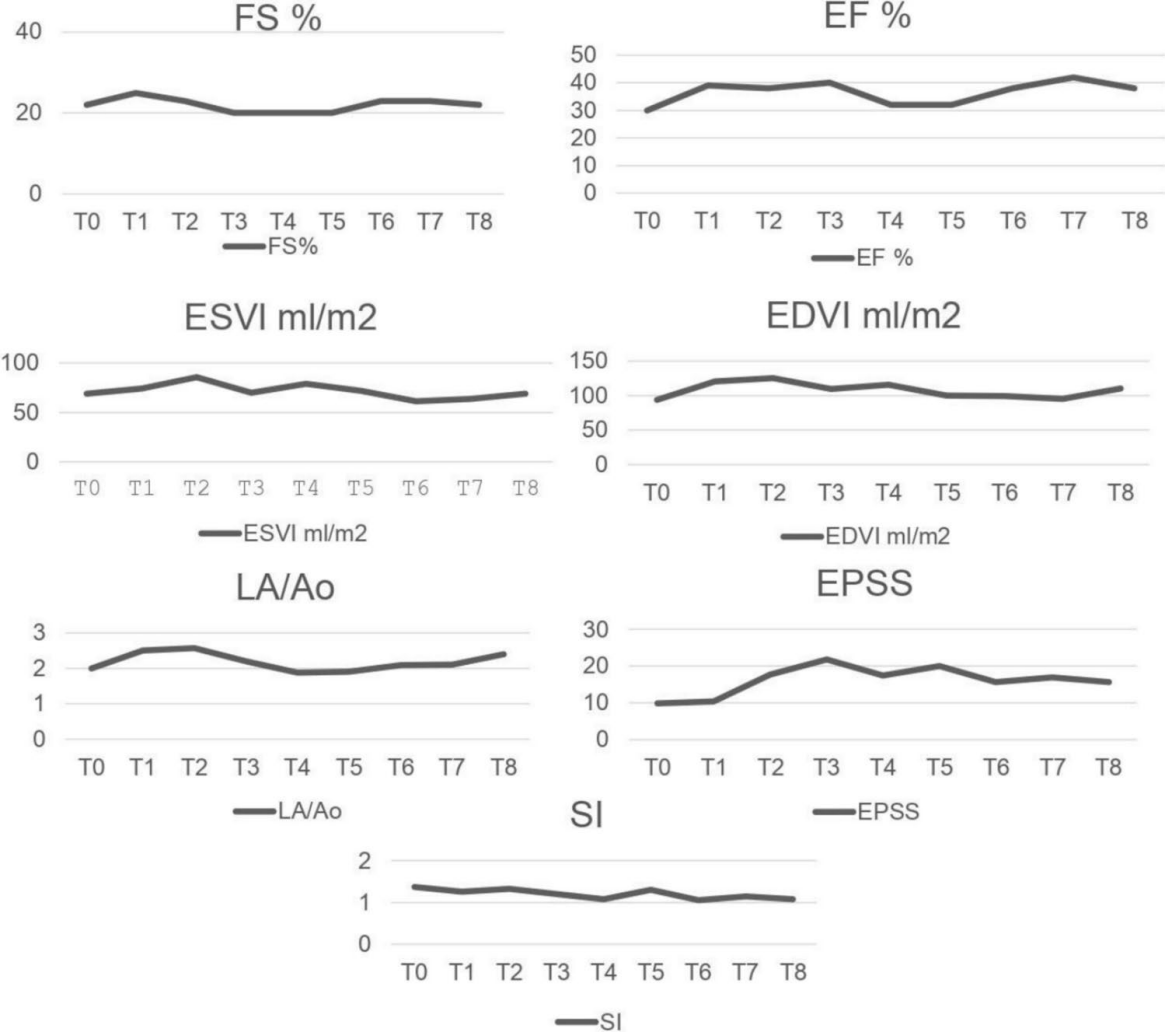
Changes of cardiac functional parameters from T0 to T8. FS, fractional shortening; EF, ejection fraction; ESVI, end-systolic volume index; EDVI, end-diastolic volume index; LA/Ao, left atrium-aorta ratio; SI, sphericity index; EPSS, mitral valve E-Point to Septal Separation

Serial monitoring was performed at 1 (T1), 3 (T2), 6 (T3), 9 (T4), 12 (T5), 18 (T6), 24 (T7) and 36 (T8) months post-procedure and included clinical, laboratory and instrumental examinations (ECG, echocardiogram). After the last follow-up, the dog was visited monthly until his death at the GP practice, thus monitoring the patient’s clinical status. The dog was clinically stable throughout the study, and he was maintained on standard medical treatment without the need for posology changes; he had normal hematobiochemical parameters and the digoxin serum concentration was within the reference range (Table 1). For echocardiographic monitoring, standard parameters like EF, ESVI and EDVI were calculated in B-mode using the Simpson method from the left apical four chamber view and FS in M-mode. Other parameters that were studied were SI, EPSS, LA/Ao and diastolic pattern, velocity of mitral and tricuspidal regurgitant jets. Echocardiographic scans and measurements were taken by the same operator (P.P.) at each time point. The echocardiographic parameters that were recorded at all the follow-up examinations are shown in Table 2. Figure 1 shows the trend over time (from T0 to T8) of the major monitored echocardiographic parameters.

**Table 1:** Haematobiochemicals parameters during follow up

CBC-Tested Values	HGB g/dL	HCT %	WBC × 1000/µL	PLT × 1000/µL	Urea mg/dL	Crea mg/dL	P^+^ mg/dL	K^+^ mEq/L	Alb g/dL	Prot.Tot g/dL	Digoxine ng/mL
Physiological Range	14,0–19,5	38,0–54,0	6,0–14,0	180–450	10,0–45,0	0,70–1,50	2,0–5,0	4,0–5,5	2,5–4,0	5,5–7,5	0,90–3,00
T0	15,5	47,3	8,5	266	72	1,52	3,5	5,0	3,0	6,3	3,10
T1	15,6	47,4	7,5	276	50	1,49	4,1	5,0	3,0	6,4	2,38
T2	15,3	43,8	7,2	213	53	1,65	3,8	5,0	2,8	6,3	
T3	16,0	45,7	5,4	233	46	1,42	3,8	4,7	3,2	6,3	1,73
T4	16,01	48	5,8	238	40	1,28	3,9	4,6	3	6,4	2,09
T5	16,1	46,9	5,5	248	63	1,46	4,3	4,8	3,1	6,4	2,2
T6	15,1	45,2	5,5	277	53	1,51	4	4,8	3,1	6,4	1,89
T7	15,9	42,5	6,33	263	73,8	0,9	3,7		3,5	6,32	
T8	16,1	44,5	8,7	339	59	1,59	4,5	5,1	2,8	6,9	0,94

*CBC* complete blood count, *HGB* hemoglobin, *HCT* hematocrit, *WBC* white blood count, *PLT* platelets, Crea creatinine, P^+^ phosphorus, K^+^ potassium, *Alb* albumin, *Tot. Prot*. total protein; T0: time of procedure; T1, T2, T3, T4, T5, T6; T7, T8: 1–3-6–9-12–18-24–36 months post procedure respectively

**Table 2:** Echocardiographic parameters during follow up

	SI	FS%	EF% Simpson	EDVI ml/m^2^	ESVI ml/m^2^	EPSS mm	LA/Ao	Mitral Regurgitation	Tricuspidal Regurgitation	Diastolic Pattern	HR bpm	Weight Kg	BSA
T0	1,37	22	30	94,1	69,1	9,9	2	Yes	No	N.D	73	82	1,9
T1	1,26	25	39	120,6	74	10,4	2,5	Yes	No	R	128	80	1,86
T2	1,32	23	38	125,6	85,4	17,7	2,57	Yes	No	R	98	80	1,86
T3	1,2	20	40	109,3	69,8	21,8	2,2	Yes	No	R	129	79	1,85
T4	1,08	20	32	115,8	79	17,4	1,87	Yes	Yes	R	100	78,5	1,84
T5	1,3	20	32	100	71,8	20	1,9	Yes	Yes	R	72	85	1,94
T6	1,05	23	38	99,32	61,18	15,6	2,09	Yes	Yes	R	80	84	1,92
T7	1,14	23	42	95,25	63,6	17	2,1	Yes	Yes	R	72	84	1,92
T8	1,08	22	38	110	68,71	15,6	2,4	yes	Yes	R	68	75	1,78

*SI* sphericity index, *FS* fractional shortening, *EF* ejection fraction, *EDVI* end-diastolic volume index, *ESVI* end-systolic volume index, *EPSS* mitral valve E-point to Septal Separation, *LA/Ao* left atrium-aorta ratio, *HR* heart rate, *BSA* body surface area; T0: time of procedure; T1, T2, T3, T4, T5, T6; T7, T8: 1–3-6–9-12–18-24–36 months post procedure respectively

**Fig. 2:**
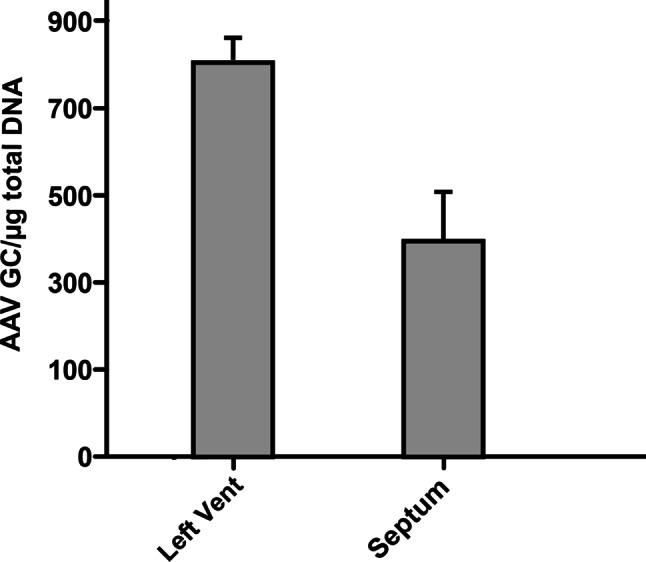
Quantification AAV genome copies in post-mortem myocardial tissue

EF initially tended to increase, then decreased and increased again until it exceeded the value found at T0 (from 30% at T0 to 38% at T8). Other parameters of left ventricular function, such as FS, did not change much over time (FS: 22% at T0; 22% at T8). Throughout the study period, there was little change in the other morphological and pathological progression parameters: LA/Ao = 2 at T0 and 2.1 at T8, and ESVI from 69.1 at T0 to 68.7 at T8. EPSS initially tended to increase, reaching a peak at T3 (21.8 mm), and then returned to lower levels. EDVI showed a more variable trend, but a mild difference was found from 94.1 ml/m^2^ at T0 to 110 ml/m^2^ at T8 (Fig. 1). A tricuspidal regurgitant jet was detected starting from T4 (9 months after the procedure). The dog survived 341 days from the last follow up and died of sudden death 1436 days after the procedure (T0). The presence of viral (AAV) DNA was detected by PCR in post- mortem heart tissue samples and AAV genome copies were quantified; results of DNA quantification are reported in Fig. 2.

## Discussion and conclusion

The administration of AAV-VEGF-B itself was well tolerated in this dog, as well as in the other dogs with DCM included in the Paradies et al. (2019) study. The dog was a 6-year-old St. Bernard, a breed at risk of developing DCM (O’Grady and O’Sullivan 2004), although there are no specific genetic factors associated with the disease in this breed. It is possible to suppose that this patient had primary DCM, albeit it could not be excluded that DCM was the result of myocarditis in this dog living outside (McCauley et al. 2020). Although less likely, TCIM could not be ruled out in this case; AF could be the consequence or the cause of the myocardial injury (Wess 2022).

The appearance of PVC during the coronary catheterization procedure has been reported in other studies and is a possible consequence of the myocardial mechanical temporary insult. In such cases, it is necessary to withdraw the catheter during the procedure (Santilli et al. 2012). The ventricular rhythm recorded a few hours post-procedure and resolved without any pharmacological intervention over the following 48 h could be the consequence of a mild and transient inflammatory reaction of the myocardium secondary to the inoculation of the viral suspension (Paradies et al. 2019). It is reasonable to think that it was not caused by VEGF-B167, since the expression of AAV-carried transgenes typically takes about 10 days (Lähteenvuo et al. 2009). The dog remained in a good and stable clinical condition throughout the study and no laboratory abnormalities were registered.

In our study, echocardiography showed no major differences over time, indicating both the absence of direct adverse cardiac events and the disease stability. The natural course of DCM is characterized by progressive worsening of the systolic dysfunction as evidenced by decreased left ventricles EF, increased ESVI, and reduced shortening along the minor and longitudinal axes of the left ventricle (Bonagura and Visser 2022). This was not the case in the present study. According to a study by Borgarelli et al. (2006), EF is an accurate index of myocardial function which significantly affects survival time. Results of echocardiographic monitoring in this dog showed that EF did not worsen as it could be expected in a progressive disease such as DCM. It is clear that statistical analysis cannot be performed on a single case and results need to be interpreted based on a single case study without appropriate controls. The fact that this patient died 1 year after T8 and 4 years post procedure (1436 days post procedure) deserves acknowledgement considering that the long-term prognosis for dogs with DCM described in the literature is much more severe. Most patients showing initial signs of CHF die within 6/7 months (Simpson et al. 2021; Vollmar et al. 2019). In more severe cases, the survival time is a few weeks/months, sometimes a more favorable clinical course is possible, with a survival of 1–2 years (Martin et al. 2010). Several factors can affect survival time in dogs with DCM including whether or not they have developed CHF or severe arrhythmias (Vollmar et al. 2019) and diet (Freid et al. 2021). It is also interesting to note that life expectancy in healthy dogs varies with breed and size (Gaar-Humphreys et al. 2022), so for a St. Bernard a lifetime of 10 years is a very good expectation.

Based on the owner’s report the dog was not showing any sign of discomfort until the night before death. One week earlier he had experienced acute symptoms of gastric distension, which were solved with a gastric tube. At autopsy, gastric torsion, concomitant chronic diseases and non-cardiac abnormalities were ruled out. Sudden death due to cardiovascular disease (i.e. arrhythmias) was likely, but death due to cardiovascular shock from acute gastric distension could not be totally ruled out as the cause of sudden death in this dog.

The presence of viral (AAV) DNA was investigated by PCR in heart tissue and AAV genome copies were quantified as an indirect evidence of transgene expression (VEGF-B) carried by the virus. To the authors’ knowledge, this is one of the rare studies in which transgene AAV persistence in the heart was documented years after delivery.

Limitations need to be highlighted in this case. First, the study reports on the monitoring and outcome after intracoronary gene delivery in a single dog. The relatively long survival time of this dog is suggestive and encouraging, but in the absence of statistical comparisons, our finding cannot yet lead to a conclusion regarding the therapeutic efficacy of AAV-VEGF-B167 in canine natural DCM. Furthermore, the dog presented here may have not had primary DCM, but possibly myocarditis or TCIM. On the other hand, there was no evidence that the transgene was still being expressed at the time of death (although post-mortem PCR documented the presence of the transgene vector in cardiac tissue). Finally, the dog remained on appropriate medical therapy throughout the study, so it is not possible to distinguish the effect of medical therapy from that of gene therapy, nor to exclude the effect of diuretics on echocardiographic parameters.

In conclusion, this clinical case, although anecdotal and affected by the limitations mentioned above, suggests beneficial effects of intracoronary delivery of AAV-VEGF-B167 in dogs with DCM, possibly due to an important cardioprotective action, as previously found in experimental models. Considering the typical time course of DCM described in the literature, a survival time of 4 years after diagnosis and gene delivery and the persistence of AAV DNA in the cardiac tissue are remarkable and encouraging findings. If additional data from other canine patients treated with AAV-VEGF-B167 will indicate a correlation between survival time and AAV abundance in cardiac tissue, more dogs will be enrolled to meet the criteria for a complete clinical study.

## Data Availability

All data are available from the authors.

## Abbreviations

VEGF: Vascular endothelial grow factor
AVV: Adeno-associated virus serotype 9
DCM: Dilated cardiomyopathy
CHF: Congestive heart failure
TICM: Tachycardia-induced myocardiopathy
EF: Ejection fraction (%)
FS: Fractional shortening (%)
ESVI: End systolic volume index (ml/m^2^)
EDVI: End diastolic volume index (ml/m^2^)
SI: Sphericity index
EPSS: E-point septal separation (mm)
LA/Ao: Left atrium/aorta ratio

## References

[CR1] Argiro A, Bui Q, Hong KN, Ammirati E, Olivotto I, Adler E (2024) Applications of gene therapy in cardiomyopathies. JACC: Heart Fail 12(2):248–260. 10.1016/j.jchf.2023.09.015 37966402 10.1016/j.jchf.2023.09.015

[CR2] Bonagura JD, Visser LC (2022) Echocardiographic assessment of dilated cardiomyopathy in dogs. J Vet Cardiol 40:15–50. 10.1016/j.jvc.2021.08.004 34750089 10.1016/j.jvc.2021.08.004

[CR3] Borgarelli M, Santilli RA, Chiavegato D, D’Agnolo G, Zanatta R, Mannelli A, Tarducci A (2006) Prognostic indicators for dogs with dilated cardiomyopathy. J Vet Intern Med 20(1):104–110. 10.1111/j.1939-1676.2006.tb02829.x 16496929 10.1892/0891-6640(2006)20[104:pifdwd]2.0.co;2

[CR4] Dukes-McEwan J, Borgarelli M, Tidholm A, Vollmar AC, Häggström J (2003) ESVC taskforce for canine dilated cardiomyopathy. Proposed guidelines for the diagnosis of canine idiopathic dilated cardiomyopathy. J Vet Cardiol 5(2):7–19. 10.1016/S1760-2734(06)70047-9 19081360 10.1016/S1760-2734(06)70047-9

[CR5] Freid KJ, Freeman LM, Rush JE, Cunningham SM, Davis MS, Karlin ET, Yang VK (2021) Retrospective study of dilated cardiomyopathy in dogs. J Vet Intern Med 35(1):58–67. 10.1111/jvim.15972 33345431 10.1111/jvim.15972 PMC7848368

[CR6] Gaar-Humphreys KR, Spanjersberg TCF, Santarelli G, Grinwis GCM, Szatmári V, Roelen BAJ, Vink A, van Tintelen JP, Asselbergs FW, Fieten H, Harakalova M, van Steenbeek FG (2022) Genetic basis of dilated cardiomyopathy in dogs and its potential as a bidirectional model. Animals 12(13):1–18. 10.3390/ani12131679 10.3390/ani12131679 PMC9265105 35804579

[CR7] Greenberg B (2017) Gene therapy for heart failure. Trends Cardiovasc Med 27(3):216–222. 10.1016/j.tcm.2016.11.001 28063800 10.1016/j.tcm.2016.11.001

[CR8] Lähteenvuo JE, Lähteenvuo MT, Kivelä A, Rosenlew C, Falkevall A, Klar J, Heikura T, Rissanen TT, Vähäkangas E, Korpisalo P, Enholm B, Carmeliet P, Alitalo K, Eriksson U, Ylä-Herttuala S (2009) Vascular endothelial growth factor-B induces myocardium-specific angiogenesis and arteriogenesis via vascular endothelial growth factor receptor-1- and neuropilin receptor-1-dependent mechanisms. Circulation 119(6):845–856. 10.1161/CIRCULATIONAHA.108.816454 19188502 10.1161/CIRCULATIONAHA.108.816454

[CR9] Li Y, Zhang F, Nagai N, Tang Z, Zhang S, Scotney P, Lennartsson J, Zhu C, Qu Y, Fang C, Hua J, Matsuo O, Fong G-H, Ding H, Cao Y, Becker KG, Nash A, Heldin C-H, Li X (2008) VEGF-B inhibits apoptosis via VEGFR-1-mediated suppression of the expression of BH3-only protein genes in mice and rats. J Clin Invest 118(3):913–923. 10.1172/JCI33673 18259607 10.1172/JCI33673 PMC2230661

[CR10] Martin MWS, Stafford Johnson MJ, Strehlau G, King JN (2010) Canine dilated cardiomyopathy: a retrospective study of prognostic findings in 367 clinical cases. J Small Anim Pract 51(8):428–436. 10.1111/j.1748-5827.2010.00966.x 20670255 10.1111/j.1748-5827.2010.00966.x

[CR11] McCauley SR, Clark SD, Quest BW, Streeter RM, Oxford EM (2020) Review of canine dilated cardiomyopathy in the wake of diet-associated concerns. J Anim Sci 98(6):1–10. 10.1093/JAS/SKAA155 10.1093/jas/skaa155 PMC7447921 32542359

[CR12] Narula J, Haider N, Virmani R, DiSalvo TG, Kolodgie FD, Hajjar RJ, Schmidt U, Semigran MJ, Dec GW, Khaw BA (1996) Apoptosis in myocytes in end-stage heart failure. New Engl J Med 335(16):1182–1189. 10.1056/NEJM199610173351603 8815940 10.1056/NEJM199610173351603

[CR13] O’Grady MR, O’Sullivan ML (2004) Dilated cardiomyopathy: an update. Vet Clin N Am Small Anim Pract 34(5):1187–1207. 10.1016/j.cvsm.2004.05.009 10.1016/j.cvsm.2004.05.009 15325477

[CR14] Paradies P, Carlucci L, Woitek F, Staffieri F, Lacitignola L, Ceci L, Romano D, Sasanelli M, Zentilin L, Giacca M, Salvadori S, Crovace A, Recchia FA (2019) Intracoronary gene delivery of the cytoprotective factor vascular endothelial growth Factor-B167 in canine patients with dilated cardiomyopathy: a short-term feasibility study. Vet Sci 6(1):23. 10.3390/vetsci6010023 30845635 10.3390/vetsci6010023 PMC6466215

[CR15] Santilli R, Bussadori C, Borgarelli M (2012) Manuale Di Cardiologia Del cane e del gatto. Elsevier Health Sciences, Italy

[CR16] Shen L, Estrada AH, Meurs KM, Sleeper M, Vulpe C, Martyniuk CJ, Pacak CA (2022) A review of the underlying genetics and emerging therapies for canine cardiomyopathies. J Vet Cardiol 40:2–14. 10.1016/j.jvc.2021.05.003 34147413 10.1016/j.jvc.2021.05.003 PMC8606013

[CR17] Simpson S, Kordtomeikel KZ, Wong S, Bennison S, El-Gendy SAA, Cobb M, Rutland SC (2021) Diagnosis, prognosis, management, treatment, research and advances in canine dilated cardiomyopathy. In: Rutland CS (ed) Canine genetics, health and medicine. 10.5772/intechopen.97682

[CR18] Vollmar C, Vollmar A, Keene BW, Fox PR, Reese S, Kohn B (2019) Dilated cardiomyopathy in 151 Irish wolfhounds: characteristic clinical findings, life expectancy and causes of death. Vet J 245:15–21. 10.1016/j.tvjl.2018.12.018 30819421 10.1016/j.tvjl.2018.12.018

[CR19] Walker AL, DeFrancesco TC, Bonagura JD, Keene BW, Meurs KM, Tou SP, Kurtz K, Aona B, Barron L, McManamey A, Robertson J, Adin DB (2021) Association of diet with clinical outcomes in dogs with dilated cardiomyopathy and congestive heart failure. J Vet Cardiol 40:99–109. 10.1016/j.jvc.2021.02.001 33741312 10.1016/j.jvc.2021.02.001

[CR20] Wess G (2022) Screening for dilated cardiomyopathy in dogs. J Vet Cardiol 40:51–68. 10.1016/j.jvc.2021.09.004 34732313 10.1016/j.jvc.2021.09.004

[CR21] Woitek F, Zentilin L, Hoffman NE, Powers J, Ottiger I, Parikh S, Kulczycki AM, Hurst M, Ring N, Wang T, Shaikh F, Gross P, Singh H, Kolpakov MA, Linke A, Houser SR, Rizzo V, Sabri A, Madesh M, Recchia FA (2015) Therapeutic intracoronary gene delivery of VEGF-B167 in a preclinical animal model of dilated cardiomyopathy. J Am Coll Cardiol 66(2):139–153. 10.1016/j.jacc.2015.04.071 26160630 10.1016/j.jacc.2015.04.071 PMC4499859

